# CircRNA_0109291 regulates cell growth and migration in oral squamous cell carcinoma and its clinical significance

**DOI:** 10.22038/IJBMS.2018.30347.7313

**Published:** 2018-11

**Authors:** Shao-Bo Ouyang, Jun Wang, Si-Yu Zhao, Xian-Hua Zhang, Lan Liao

**Affiliations:** 1 Department of Oral Prosthodontics, the Affiliated Stomatological Hospital of Nanchang University, Jiangxi Provincial Key Laboratory of Oral Biomedicine, Nanchang 330006, China; 2 Department of Oral and Maxillofacial Surgery, the Second Affiliated Hospital of Nanchang University, Nanchang 330006, China

**Keywords:** Apoptosis, CircRNA, Hsa_circ_0109291, Oral squamous cell – carcinoma, Prognosis

## Abstract

**Objective(s):**

Circular RNAs (circRNAs), a new class of non-coding RNAs, have emerged as important regulators during tumorigenesis. However, the functions of circRNAs have not been completely clarified in the progression of cancers. In our study, a novel circRNA hsa_circ_0109291 was investigated in oral squamous cell carcinoma (OSCC) tissues and cell lines.

**Materials and Methods:**

The expression profile of circRNAs in OSCC tumor tissues was performed by high-throughput sequencing. The CCK-8 wound healing and apoptosis assay were measured in OSCC cell lines after transfection with si-0109291 or si-NC.

**Results:**

We discovered that hsa_circ_0109291 was significantly increased in OSCC tissues and cell lines compared with their corresponding control group. Knockdown of hsa_circ_0109291 inhibited proliferation and migration of OSCC cell lines *in vitro*. In addition, inhibition of hsa_circ_0109291 dramatically induced apoptosis of OSCC cells. We further found that high hsa_circ_0109291 levels in OSCC patients resulted in a poorer prognosis than in patients with low hsa_circ_0109291 levels.

**Conclusion:**

These findings indicated that hsa_circ_0109291 correlated with the progression of OSCC and might be a new therapeutic target for the treatment of OSCC.

## Introduction

Squamous cell carcinoma (OSCC) is one of the frequently occurring malignancies, with approximately 540,000 newly diagnosed cases annually and 5-year survival rate of less than 50% (1). The major reasons for the low survival rates of OSCC are lymph node metastasis and local or regional recurrence (2). Alcohol consumption, tobacco use, and human papillomavirus (HPV) infection are widely recognized as the main risk factors of OSCC (3). OSCC is histopathologically characterized by keratinization and squamous differentiation (4), which are complex pathological processes and may be related to the change of a variety of biological molecules such as growth factors (5), microRNAs (miRNAs) (6, 7) and long non-coding RNAs (lncRNAs) (8, 9), suggesting non-coding RNAs may play a crucial role in these process. 

Circular RNAs (circRNAs) as a new class of non-coding RNAs are characterized by a covalently closed continuous loop, without 5’ to 3’ polarity and polyadenylated tail that endow the stable structure for circRNAs (10). CircRNAs are originally reported as the splicing products of endogenous RNA and considered as the byproducts of splicing errors (11). With the in-depth study of their pathophysiological features, circRNAs are widely expressed in mammals and participate in the pathogenesis of several types of diseases such as myocardial fibrosis (12), Alzheimer’s disease (13), preeclampsia (14), and osteoarthritis (15). Recent studies have revealed that circRNAs perform their function by acting as miRNA sponges (16, 17). It is well known that circRNA ciRS-7 functions as a miRNA-7 sponge and contains more than 70 binding sites for miR-7 (16). Moreover, circ-HIPK3 (18), circ-ITCH (19), and circMTO1 (20) are also able to act as miRNA sponges. Accumulating evidence demonstrates that circRNA targeting to miRNA, as a classical signal transduction pathway, participates in the initiation and development of cancer, including breast cancer (21), gastric cancer (22), colorectal cancer (23), and hepatocellular carcinoma (20). Recently, a handful of studies have revealed that circRNAs are involved in the development and progression of OSCC (24, 25). However, the potential regulatory mechanisms of circRNAs in OSCC have not been completely clarified, and their clinical and prognostic significance has yet to be reported.

In our study, high-throughput sequencing analysis was carried out to elaborate the differentially expressed circRNAs in OSCC and normal tissues, and the results demonstrated that hsa_circ_0109291 was significantly higher in OSCC tissues than in adjacent normal tissues. In addition, we examined the correlation between hsa_circ_0109291 expression in OSCC tissues and clinical outcomes. Furthermore, the association of hsa_circ_0109291 and cell proliferation, migration, and apoptosis were performed in OSCC cell lines. 

## Materials and Methods

***Patients and specimens***

Fifty-one pairs of OSCC tissues and adjacent normal tissues were collected from the Affiliated Stomatological Hospital of Nanchang University (Nanchang, China) between January 2014 and June 2017. All of OSCC patients were recruited according to the histopathological evaluation without radiotherapy or chemotherapy before a surgical operation. All OSCC tissues and normal tissues were immediately stored in liquid nitrogen after surgical operation. Written informed consent was obtained from the patients. This study was approved by the Ethics Committee of the Affiliated Stomatological Hospital of Nanchang University (Nanchang, China).

**Figure 1:**
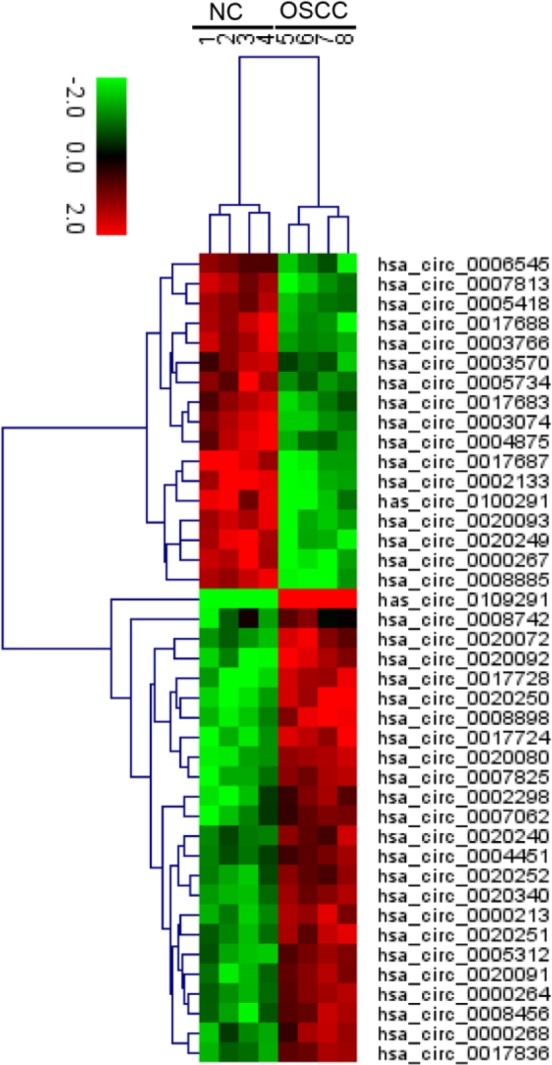
CircRNAs expression profile in oral squamous cell carcinoma tumor tissues. OSCC-related circRNAs were selected out based on FDR ≤ 0.001 and fold change ≥ 2 or fold change ≤ 0.5. 41 circRNAs were differentially expressed between OSCC tissues and normal tissues. The heat map showed the 41 differentially expressed circRNAs, red indicating high expression and green indicating low expression

**Figure 2:**
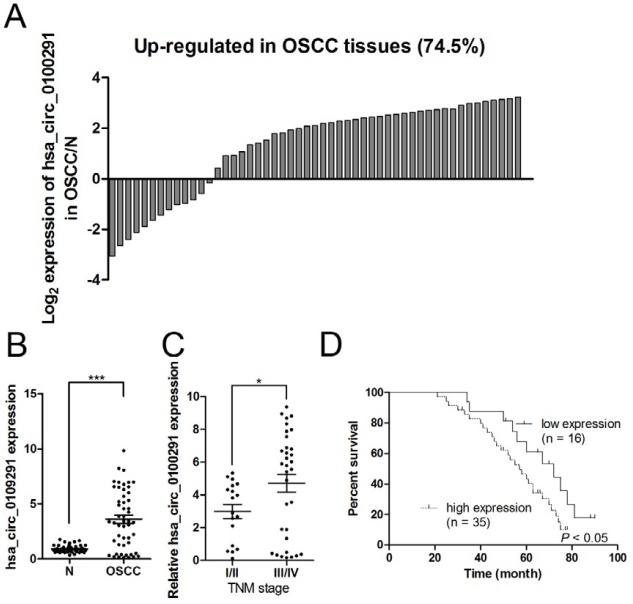
Hsa_circ_0109291 expression was up-regulated in oral squamous cell carcinoma tumor tissues. Hsa_circ_0109291 expression was measured in 51 tumor tissues and matched adjacent non-tumorous tissues from OSCC patients, and the fold change was calculated (A and B). Hsa_circ_0109291 correlated to the advanced TNM stage (C). Kaplan-Meier survival curve was used to evaluate whether hsa_circ_0109291 expression levels were associated with overall survival in patients with OSCC (D). * P<0.05, *** *P*<0.001

**Figure 3:**
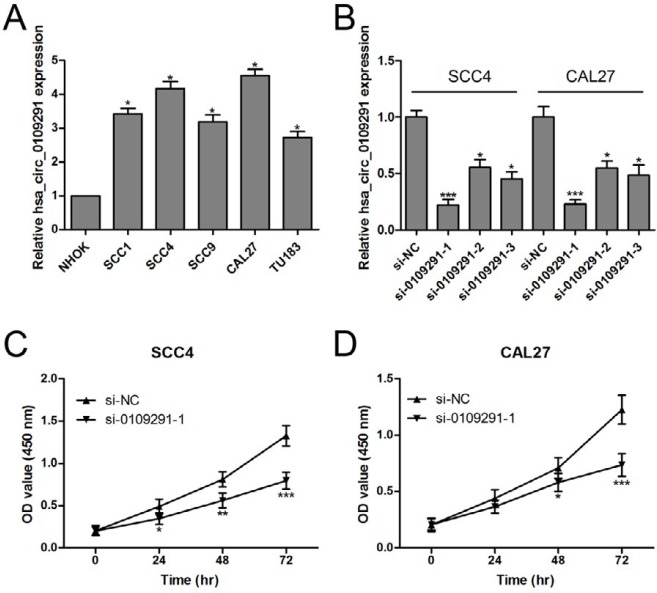
Inhibition of hsa_circ_0109291 suppresses oral squamous cell carcinoma cell growth *in vitro*. Relative expression of hsa_circ_0109291 in NHOK cells and OSCC cell lines was measured by RT-qPCR (A). The efficiency of si-RNA to inhibit hsa_circ_0109291 expression was verified by RT-qPCR (B). The cell viability of SCC4 (C) and CAL27 (D) was measured by CCK-8 assay after transfection with si-0109291-1 and si-NC for 0-72 hr. * *P*<0.05, ** *P*<0.01 and *** *P*< 0.001

**Figure 4:**
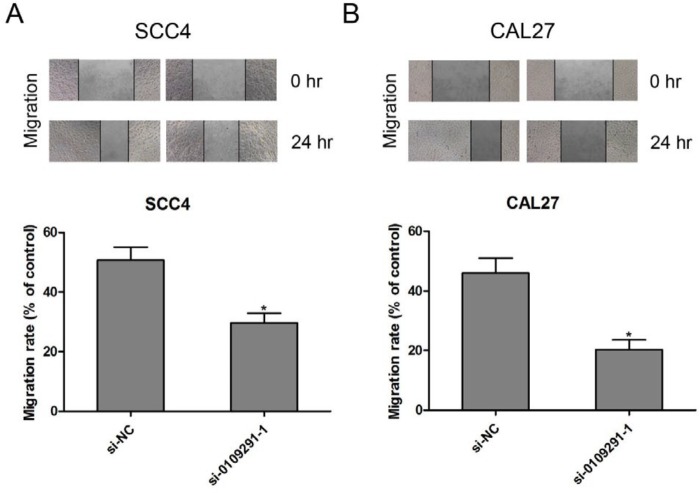
Inhibition of hsa_circ_0109291 suppresses oral squamous cell carcinoma cell migration *in vitro*. After transfection with si-0109291-1 and si-NC, SCC4 (A) and CAL27 (B) cell migration was determined by wound healing assay for 24 hr. * *P*<0.05

**Figure 5:**
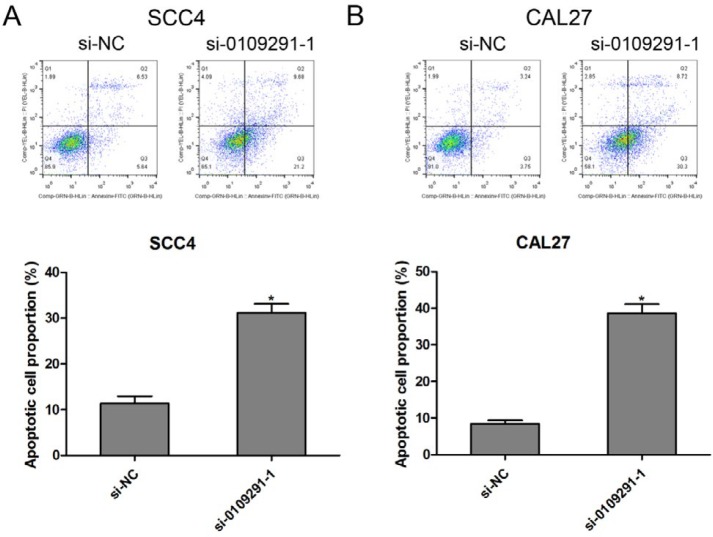
Inhibition of hsa_circ_0109291 induced oral squamous cell carcinoma cell apoptosis *in vitro*. After transfection with si-0109291-1 and si-NC, SCC4 (A) and CAL27 (B) cell apoptosis was detected by flowcytometry for 24 hr. * *P*<0.05

**Figure 6:**
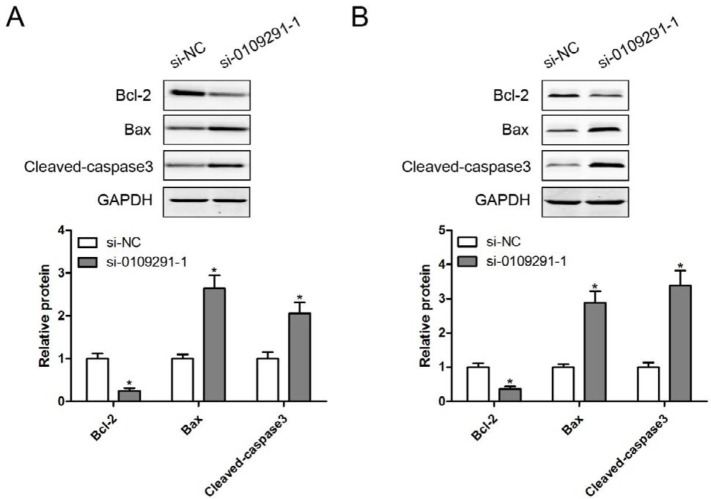
Inhibition of hsa_circ_0109291 regulated apoptosis-related protein expression. After transfection with si-0109291-1 and si-NC, protein expression of Bcl-2, Bax, and Cleaved-caspase3 was measured by Western blotting in SCC4 (A) and CAL27 (B) cells for 24 hr. * *P*< 0.05

***Cell culture***

Normal human oral keratinocyte (NHOK) and five OSCC cell lines (SCC-1, SCC-4, SCC-9, CAL27, and TU183) were purchased from the Cell Bank of Type Culture Collection of Chinese Academy of Sciences (Shanghai, China). Cells were cultured in Dulbecco’s modified Eagle’s medium (DMEM; Invitrogen, Carlsbad, CA, USA) with 5% fetal bovine serum (Thermo Scientific HyClone, Beijing, China), 5% CO_2_, and 95% air atmosphere in a humidified incubator (Thermo, USA). 

***High-throughput sequencing***

CircRNAs high-throughput sequencing analysis was performed as described previously (26). According to Memczak *et. al.* methods (27), the clean reads were aligned to the reference genome by Bowtie2 (http://bowtie-bio.sourceforge.net/bowtie2/manual.shtml). For unmapped reads, the junctions were picked out using the back-splice algorithm. CircRNAs levels were calculated using “Mapped back-splicing junction reads per million mapped reads” (RPM). 

***Cell transfection***

The small interfering RNA (si-RNA) was synthesized by GenePharma Co., Ltd. (Shanghai, China) to inhibit the expression of hsa_circ_0109291 in SCC4 and CAL27 cells. The targeted sequence of the functional si-0109291-1, -2, or -3 were 5’-AATCCCCAGGAGACGTTGACA-3’, 5’-CCCCAGGAGACGTTGACATTT-3’, or 5’-ATGAATCCCC-

AGGAGACGTTG-3’, respectively. Three si-RNAs were transfected into SCC4 and CAL27 cells using Lipofectamine 2000 (Invitrogen) according to the manufacturer’s protocol. Finally, si-0109291-1 was selected and was used in proliferation, migration, apoptosis, and apoptosis-related protein assays. All experiments were repeated three times.

***CCK-8 assay***

The cell viability of SCC4 and CAL27 cells (1×10^4^) was detected using a CCK-8 assay kit (Dojindo, Japan). The absorbance was measured at 450 nm using a SpectraMax M5 plate reader (Molecular Devices, USA). The CCK-8 proliferation assay was performed as previously described (4). 

***Wound healing assay***

SCC4 and CAL27 cells (2×10^5^) were trypsinized and reseeded in new 6-well plates. With 24 hr incubation, the confluent cells monolayers were scratched with a 10 μl sterile pipette tip. The wound healing assay was determined as previously described (28). The migration rate was monitored by the inverted microscope (Olympus, Japan). 

***Flow cytometry for apoptosis***

The cell apoptosis assay was performed as previously described (25). Annexin V-FITC apoptosis detection kit was purchased from Invitrogen (Carlsbad, Calif, USA). The apoptosis rate was analyzed using A flowcytometer (BD Biosciences, Franklin Lakes, NJ, USA) and CELL Quest 3.0 software (BD Biosciences). 

***Total RNA extraction***

Total RNA was extracted from the OSCC tissues, normal tissues, and cell lines using Trizol reagent (Invitrogen, Carlsbad, CA, USA) and preserved at -80 ^°^C until use. 

***RT-qPCR***

Moloney Murine Leukemia Virus Reverse transcriptase (Promega Corporation, Madison, WI, USA) was used to synthesize cDNA. Divergent primers were designed and used to measure circRNA expression using an ABI7300 System (Applied Biosystems, Foster City, CA, USA) with SYBR Select Master Mix (Applied Biosystems). Glyceraldehyde-3-phosphate dehydrogenase (GAPDH) served as an internal control gene to normalize the expression of hsa_circ_0109291. The PCR primers were used as follows: hsa_circ_0109291, forward 5’-TGCTGTCTCTAAGCAAGACCC-3’ and reverse 5’-AGGGTTCAGGCATTCCCACT-3’; GAPDH, forward 5’-GCACCGTCAAGCTGAGAAC-3’, and reverse 5’-TGGTGAAGACGCCAGTGGA-3’. The 2^-ΔΔCt^ method was used to calculate the expression of hsa_circ_0109291 (29).

***Western blotting***

Total protein in SCC4 and CAL27 cells was extracted using RIPA Lysis Buffer (Beyotime Institute of Biotechnology, Haimen, China). 30 μg of total protein were separated by 10% SDS-PAGE gel and transferred to nitrocellulose membranes (Bio-Rad Laboratories, Inc., Hercules, CA, USA). Primary antibodies Bcl-2 (cat.no: sc-56015, dilution, 1:1,000) and Bax (cat.no: sc6236, dilution, 1:1,000) were purchased from Santa Cruz Biotechnology (Santa Cruz, CA, USA). Cleaved-Caspase3 (cat.no: 9661, dilution, 1: 1,000) was purchased from Cell Signaling Technology, Inc. (Massachusetts, USA). Then, the membranes were incubated with secondary antibody (cat.no: sc-516102; dilution: 1:10,000; Santa Cruz Biotechnology) at room temperature for 2 hr. GAPDH (Cat. no: 2118; dilution: 1:2,000; Cell Signaling Technology, Inc.) served as an internal control gene to normalize the protein expression.

***Statistical analysis***

Data were analyzed using GraphPad Prism Version 7.0 (GraphPad Software, Inc., La Jolla, CA, USA) and presented as the mean ± standard deviation (SD). Student *t*-test was used to analyze two-group differences. Inter-group differences were analyzed by one-way analysis of variance, followed by Tukey’s *post hoc* analysis. Survival analysis was performed using the Kaplan-Meier method. *P*-values less than 0.05 was considered to indicate a statistically significant difference.

## Results

***Expression pattern of circRNAs in OSCC***

The expression profiles of circRNAs in 4 pairs of OSCC and corresponding adjacent non-cancerous tissues were detected using circRNAs high-throughput sequencing analysis. Based on FDR ≤ 0.001 and fold change ≥ 2 or fold change ≤ 0.5, the candidate circRNAs were filtered out, and the results demonstrated that 41 circRNAs were selected out, among which 17 circRNAs and 24 circRNAs were up-regulated and down-regulated, respectively (Figure 1). Among these up-regulated circRNAs, the fold change of hsa_circ_0109291 was up-regulated by nearly 5-fold and was the highest. To verify the sequencing results, RT-qPCR assay was performed in 51 pairs of OSCC and normal tissues. In accordance with the sequencing results, RT-qPCR assay demonstrated that hsa_circ_0109291 was up-regulated in 38 of 51 OSCC cases (74.5%) and was significantly increased (approximately 4-fold) in OSCC tissues compared with adjacent normal tissues (Figures 2A and 2B). Therefore, we focused on hsa_circ_0109291 in our study. 

To evaluate the clinical significance of hsa_circ_0109291 in OSCC patients, we found that hsa_circ_0109291 was positively correlated with the TNM stage in OSCC patients (Figure 2C). In addition, patients with low hsa_circ_0109291 expression (n = 16) had a better survival prognosis than those OSCC patients (n = 35) with high hsa_circ_0109291 levels in OSCC tissues (Figure 2D). 

***Inhibition of hsa_circ_0109291 suppresses OSCC cells growth in vitro***

The expression levels of hsa_circ_0109291 in NHOK and OSCC cell lines (SCC1, SCC4, SCC9, CAL27, and TU183) were detected by RT-qPCR. As expected, hsa_circ_0109291 was dramatically up-regulated in all OSCC cell lines as compared with NHOK cells. Intriguingly, hsa_circ_0109291 expression was markedly higher in SCC4 and CAL27 cells than in SCC1, SCC9, or TU183 (Figure 3A). Hence, SCC4 and CAL27 cells were focused on in our further experiments. After transfection with control si-RNA or si-RNAs against hsa_circ_0109291 (si-0109291-1, si-0109291-2, and si-0109291-3), the RT-qPCR results indicated that the inhibition efficiency of si-0109291-1 was markedly higher than si-0109291-2 and si-0109291-3 in SCC4 and CAL27 cells (Figure 3B). Introduction of si-0109291-1 notably suppressed SCC4 (Figure 3C) and CAL27 (Figure 3D) cells proliferation.

***Inhibition of hsa_circ_0109291 suppresses OSCC cells migration and induces apoptosis in vitro***

The effect of si-0109291-1 on OSCC cell migration was determined by wound healing assay. Compared with the si-NC group, the wound closing was significantly blunted in si-0109291-1 transfected SCC4 (Figure 4A) and CAL27 (Figure 4B) cells, suggesting that inhibition of hsa_circ_0109291 can block OSCC cell migration in vitro. Moreover, we performed flow cytometry analysis to further evaluate whether si-0109291-1 regulates OSCC cell proliferation by altering apoptosis. SCC4 and CAL27 cells were treated with si-NC or si-0109291-1 for 24 hr, the rate of apoptosis was significantly increased when SCC4 (Figure 5A) and CAL27 (Figure 5B) cells were transfected with si-0109291-1 compared with si-NC. Furthermore, we also found that hsa_circ_0109291 knockout significantly decreased the Bcl-2 protein expression and increased the protein expression of Bax and cleaved-caspase3 in SCC4 (Figure 6A) and CAL27 (Figure 6B) cells. 

## Discussion

The present study showed robust circRNA expression of hsa_circ_0109291 in OSCC, and silencing of hsa_circ_0109291 dramatically inhibited growth and migration and induced apoptosis in OSCC cells. These findings indicated that hsa_circ_0109291 might play an oncogenic role in the tumorigenesis of OSCC. Therefore, we deduced that hsa_circ_0109291 might be a potential therapeutic target for the clinical management of OSCC. 

CircRNAs as a subset of non-coding RNAs are abundant in human cells and have recently emerged as a novel regulator of gene expression in a variety of cancers, including oral cancer (30). Based on high throughput microarray assay, circDOCK1 (has_circ_100721) is significantly up-regulated in OSCC tissues and suppresses OSCC cell apoptosis via regulating the miR196a5p/BIRC3 signaling pathway (31), suggesting that circDOCK1 may function as a competing endogenous RNA (ceRNA) in OSCC carcinogenesis. Recently, a circRNA microarray analysis indicates that hsa_circ_100290 is up-regulated in OSCC tissues and cell lines and induces CDK6 expression, while inactivation of circRNA_100290/CDK6 signaling can inhibit OSCC cell proliferation by releasing miR-29 expression (24). In our study, a new functional type circRNA, hsa_circ_0109291, was identified and found that overexpression of hsa_circ_0109291 was strongly linked to poor prognosis, hsa_circ_0109291 loss-of-function could significantly inhibit OSCC cell growth and induce apoptosis. 

The further investigation found that the genomic length of hsa_circ_0109291 is 726 bp, and the spliced length is 226 bp, which is located in chr19:21280990-21281716, and its associated-gene symbol is zinc finger protein 714 (ZNF714; http://www.circbase.org/). ZNF proteins are a class of transcription factors that regulate multiple genes expression in transcriptional levels (32). Increasing evidence reveals that ZNF proteins have a double effect on cancer progression, including carcinogenesis and tumor suppressors (33). A recent study reveals that overexpressed ZNF703 contributes to cell proliferation and metastasis in OSCC (34). ZNF510 is significantly higher in OSCC tissues than in the OSCC free control tissues and has been discovered as a novel biomarker for detection of OSCC in the early stages (35). In contrast, ZNF750 as a tumor suppressor inhibits OSCC cell invasion and migration, suggesting that ZNF750 may inhibit cell metastasis during OSCC progression (36). Previous studies show a great interest in the underlying mechanism of ZNF proteins in the initiation and development of cancer, ZNF proteins mainly activate or suppress downstream genes via recruiting different interacting partners (33). Interestingly, cancer-related miRNAs, including miR-31, -199a-3p, and -525-3p can be regulated by ZNF proteins in a variety of tumors (33, 37), indicating that non-coding RNAs as post-translational regulatory mechanisms are involved in the development of cancers by regulating ZNF proteins expression. The present study indicated that circRNAs might be associated with ZNF proteins-related tumorigenesis, which provides a new theoretical insight to explore the underlying molecular mechanisms in OSCC. 

Recent studies suggest that circRNAs can function as potential molecular markers of cancer to support diagnosis, which are more stable than other non-coding RNAs *in vivo,* due to their resistance to RNase activity (17, 30). In gastric cancer, hsa_circ_0000190 is found to be a potential biomarker and has better sensitivity and specificity than classic biomarkers, such as carcinoembryonic antigen (CEA) and CA19-9 (38). Overexpression of circRNA_100876 is positively related to lymph node metastasis and TNM stage in non-small cell lung cancer (NSCLC), indicating that it may be a potential prognostic marker for NSCLC (39). Hsa_circ_0001649, 0005075, and 0004018 are identified as valuable biomarkers for hepatocellular carcinoma diagnosis and prognosis (40-42). In our work, hsa_circ_0109291 was significantly up-regulated in OSCC and correlated with poor prognosis; further studies are required to confirm the diagnostic significance of hsa_circ_0109291 as a blood-based marker for OSCC. 

## Conclusion

Taken together, both circRNA high-throughput sequencing and RT-qPCR showed hsa_circ_0109291 was markedly increased in OSCC tissues. Hsa_circ_0109291 might exert regulatory functions in OSCC cell growth, migration, and apoptosis indicating that hsa_circ_0109291 might play a crucial role in OSCC tumorigenesis. These findings suggest that hsa_circ_0109291 can serve as a potential therapeutic target for the treatment of OSCC and may be a potential biomarker for OSCC diagnosis and prognosis.
